# Experienced barriers in shared decision‐making behaviour of orthopaedic surgery residents compared with orthopaedic surgeons

**DOI:** 10.1002/msc.1390

**Published:** 2019-02-27

**Authors:** Jeroen K.J. Bossen, Trudy van der Weijden, Erik W. Driessen, Ide C. Heyligers

**Affiliations:** ^1^ School of Health Professions Education Maastricht University Maastricht the Netherlands; ^2^ Department of Family Medicine, School CAPHRI, Care and Public Health Research Institute Maastricht University Medical Centre+ Maastricht the Netherlands; ^3^ Department of Orthopaedic Surgery and Traumatology Zuyderland Medical Centre Heerlen the Netherlands

**Keywords:** attitude, medical education, orthopaedics, osteoarthritis, shared decision‐making, theory of planned behaviour

## Abstract

**Introduction:**

In shared decision‐making (SDM), physicians encourage the patient to participate in the care process. The theory of planned behaviour describes that behaviour is dependent on intention. In its turn, intention is explained by attitude, subjective norm and perceived behavioural control. In orthopaedics, little is known about current SDM behaviour and how to promote it.The aim of the present study was to gain insight into the SDM behaviour of orthopaedic residents and supervisors by measuring levels of intention, attitudes, subjective norms and perceived behavioural control. Furthermore, we aimed to determine the predictors of intention for SDM.

**Methods:**

A questionnaire survey study was conducted among orthopaedic surgeons and residents working in the care of hip and knee osteoarthritis, to determine their intentions, attitudes, subjective norms and perceived behavioural control regarding SDM.

**Results:**

Of the 385 physicians approached, 71 residents and 64 orthopaedic surgeons participated. Residents and the supervisors alike had positive intentions regarding SDM. Intention for SDM behaviour was explained by attitude, subjective norm and perceived behavioural control, with perceived behavioural control having the strongest association. In residents, the intention to engage in SDM was more hampered by a lower level of perceived behavioural control than in surgeons.

**Conclusions:**

Physicians are willing to perform SDM and consider SDM as favourable in the orthopaedic clinic. The implementation of SDM is mainly hampered by experienced barriers that they cannot control. These findings underline the importance of incorporating SDM in the curriculum of postgraduates. Possibilities for efficient SDM implementation should be explored, to overcome perceived barriers.

## 1 INTRODUCTION

Many efforts are made to enhance patient participation, but it remains difficult to direct the behaviour of physicians to more patient‐centred practice (Härter, Moumjid, Cornuz, Elwyn, & van der Weijden, 2017). Patient participation in clinical decision‐making is embodied in the concept of shared decision‐making (SDM), in which the patient and physician share responsibility in this process (Elwyn et al., 2012; Härter et al., 2017; Légaré et al., 2014). To improve SDM, clinicians increasingly use supporting programmes (van der Weijden et al., 2017), such as the Ask 3 Questions campaign and patient decision aids (Elwyn et al., 2016; Shepherd et al., 2016). However, some authors warn against the implementation of patient decision aids without appropriate education for physicians because this might lead to the use of patient decision aids without coaching patients in the decision‐making process, or even to patients feeling burdened with decision‐making responsibilities (van der Weijden et al., 2017). Little is known about the current SDM behaviour of physicians and how to promote it (Légaré et al., 2014).

To explain behaviour in relation to health outcomes, various studies have used the theory of planned behaviour (TPB) (Ajzen, 1991). The theory was also used to design theory‐based interventions to change clinicians’ behaviour (Godin, Bélanger‐Gravel, Eccles, & Grimshaw, 2008). The key component in the TPB model is behavioural intention, which is related to actual behaviour (Ajzen, 1991; Thompson‐Leduc, Clayman, Turcotte, & Légaré, 2015). Behavioural intention is determined by three independent variables: (a) attitude—that is, the degree to which a person has a favourable or unfavourable evaluation of the behaviour of interest; (b) subjective norm—that is, a person's beliefs about whether peers and people of importance think that he or she should engage in the behaviour; and (c) perceived behavioural control—that is, the perceived ability to perform a behaviour and to deal with anticipated obstacles (Ajzen, 1991).

In 2014, a review article on 20 studies that used the TPB to assess SDM behaviour in health professionals observed that these three variables predicted intention for SDM or actual SDM behaviour (Thompson‐Leduc et al., 2015). Although there was a large variance, the intention to engage in SDM was most strongly associated with subjective norm. Although intention to engage in SDM is predicted by all three predictors of intention, subjective norm was most strongly associated. A possible explanation for this finding is that SDM is a direct social interaction between the physician and the patient, and hence its favourable or unfavourable outcome is perceived to be highly dependent on issues that are not under the physician's control, such as the patient's competencies and contextual barriers and facilitators (Thompson‐Leduc et al., 2015). Another explanation for subjective norm being dominant is that there is currently a strong social movement in favour of patient‐centred care (Thompson‐Leduc et al., 2015).

Apparently, the intention to engage in SDM varies between settings and disciplines. The extent to which physicians use SDM in practice is influenced by individual and organizational factors (Alguera‐Lara, Dowsey, Ride, Kinder, & Castle, 2017; Farrelly et al., 2016; Gravel, Légaré, & Graham, 2006; Hofstede et al., 2013). For instance, qualitative research on the care of patients with herniated back pain showed that physicians had negative attitudes towards SDM. They found it important to express their views on the available treatment options and were afraid that SDM would result in a choice of treatment which they did not consider appropriate (Hofstede et al., 2013). Besides negative attitudes, other important barriers to physicians performing SDM are organizational obstacles, such as lack of time (Légaré, Ratté, Gravel, & Graham, 2008).

Little is known about differences in SDM behaviour between orthopaedic surgery residents (i.e., physicians participating in a training programme for this medical speciality) and orthopaedic surgeons. This information is relevant as attitudes and educational needs may differ between these groups, and educational programmes need to be tailored towards these needs. A Swiss study showed that residents had more negative attitudes towards SDM than their teachers (van der Horst, Giger, & Siegrist, 2011). The authors speculated that these negative attitudes might be caused by the lack of structural education in SDM communication in residency programmes (Légaré et al., 2008), and called for more SDM education in these programs (van der Horst et al., 2011).

The aim of the present study was to gain insight into the SDM behaviour of orthopaedic residents and their supervisors, in order to be able to improve the design of postgraduate educational programmes on SDM. Therefore, we assessed the levels of intention, attitudes, subjective norms and perceived behavioural control of Dutch orthopaedic surgeons and residents concerning SDM in the daily care of patients with hip or knee osteoarthritis.

## 2 METHODS

### 2.1 Study design

We performed a survey study among an unselected group of all Dutch orthopaedic surgeons and orthopaedic surgery residents who treat patients with hip and knee osteoarthritis. We obtained approval from the Medical Ethical Committee Zuyd (study number 16‐N‐195).

### 2.2 Population and procedure

We invited orthopaedic surgeons (staff physicians) and residents in training for orthopaedic surgery who treat patients with hip and knee osteoarthritis in the Netherlands. We received the contact information for these physicians from the Dutch Orthopaedic Association. We addressed the physicians and residents by email. After the first invitation, we sent two reminders to complete the survey. Informed consent was obtained before completion of the questionnaire. The questionnaire consisted of 36 questions, and participating physicians needed 10–15 min to complete this survey electronically.

### 2.3 Responder analysis

We analysed the professional role of the physicians who did not respond to our survey. The distribution of the professional roles of the nonresponders did not differ significantly from those of the participating physicians, as 56% of the nonresponders were residents and 44% orthopaedic surgeons. No other variables were recorded about nonresponding physicians.

### 2.4 Measurement

We developed a questionnaire to measure the intention to engage in SDM and the determinants of this intention. The questionnaire was based on a manual for developing TPB questionnaires (Francis et al., 2004). To our knowledge, no validated questionnaire has previously been published to determine the SDM behaviour of physicians working in orthopaedics, based on the TPB. Our questionnaire contained questions about behavioural intention and about the three main determinants of intention: attitude, subjective norm and perceived behavioural control.

From the literature, we selected factors for attitudes, subjective norm and perceived behavioural control that are associated with SDM behaviour (Alguera‐Lara et al., 2017; Farrelly et al., 2016; Geiger, Liethmann, Reitz, Galalae, & Kasper, 2017; Gravel et al., 2006; Rusiecki et al., 2018; Sanders et al., 2017). All listed authors were consulted, and a selection from these factors was made for the items of the questionnaire.

Six items were constructed for attitude, five for subjective norm and seven for perceived behavioural control. Each item consisted of two sub‐items. The first sub‐item was the selected attitude, subjective norm factor or factor of perceived behavioural control. The second sub‐item indicated the importance or relevance of the specific first sub‐item for the participant. More specifically, for the items on attitude, the first sub‐item was about the selected belief or attitude regarding SDM. The second sub‐item was about the corresponding positive or negative judgement about this attitude. For subjective norm, each first sub‐item scored a particular belief on SDM held by other people or groups. The second sub‐item scored how much the belief of these people or groups influenced the participant's behaviour. For perceived behavioural control, each first sub‐item scored a perceived barrier or facilitating factor for performing SDM, and the second sub‐item scored how much control the participant had over these factors.

1. Answers were given on a 7‐point Likert scale with a consistent direction of effect. To score the items, the first sub‐item was multiplied by the second sub‐item, and the square root of this score is taken as the final score for that particular item. Scores ranged from 1 to 7, with a high score representing high intention, positive attitude, high subjective norm or high perceived behavioural control (see Supporting Information Table S1 for the questions measuring attitude, subjective norm and perceived behavioural control). Demographic data were collected with questions regarding age, professional role (resident or orthopaedic surgeon), type of hospital (academic or peripheral hospital) and gender.

We pilot‐tested the questionnaire with three orthopaedic residents and two orthopaedic surgeons, who were not part of the research team, to ensure its usability and to identify points that needed clarification; this resulted in some small adjustments to the questionnaire.

### 2.5 Analysis

The manual we used to develop the questionnaire advised the use of a sample size of 80 participants, based on an effect size of 0.3 points on the questionnaire (Francis et al., 2004). The population of interest consisted of 395 persons, so we expected that this sample size would be achieved.

We tested the internal reliability of the questionnaire. We calculated Cronbach's alpha for the three dimensions. The Cronbach's alpha values for the subscales of attitude, subjective norm and perceived behavioural control were 0.87, 0.55 and 0.84, respectively. No questions were deleted after this analysis, as this would not have increased the Cronbach's alpha value.

The main outcome measures were not normally distributed at final evaluation, and therefore we used nonparametric tests for our analyses. For the sums of the scores for attitude, subjective norm and perceived behavioural control, we calculated mean scores, and for the question about intention we used a median as a measure of centre. We tested the differences between orthopaedic surgeons and residents in the scores of the dimensions of the TPB using the Mann–Whitney U test. Age was transformed from an ordinal variable (7 age groups) to a dichotomous variable. The cut‐off point was 35 years of age, with the younger group constituting 67% of the participants, and the older group 33%.

In the bivariate analyses, we calculated how attitude, subjective norm and perceived behavioural control were correlated with intention, using Spearman's rho correlations. All parameters with a *p*‐value lower than 0.10 in the bivariate analysis were entered into multivariate linear regression analysis, with intention as the dependent outcome. We used the enter method in our regression analyses. We assessed multicollinearity in the model, using the variance inflation factor (VIF). The VIF was found to be satisfactory (mean VIF = 1.66).

We reported the baseline characteristics of the participating physicians, and performed a nonresponder analysis of our data on the nonresponding physicians.

## 3 RESULTS

Between April 2017 and June 2017, we sent the survey to 395 physicians, 46% of whom were orthopaedic surgeons and 54% were residents. Of these, 135 (34%) completed the questionnaire. Most physicians were aged between 31 and 35 years (47%) and were male (84%). Of the responders, 48% were orthopaedic surgeons and 52% were residents.

### 3.1 Outcome measures

Both the residents and the orthopaedic surgeons scored highly on intention for SDM behaviour, with a median of 6.0 on the 7‐point Likert scale questionnaire (see Table 1).

**Table 1:** Scores questionnaire TPB of residents and orthopaedic surgeons *n* = 13

	Total group ( *n* = 135) Median	Residents(*n* = 71) Median	Orthopaedic surgeons (*n* = 64) Median	*p*‐Value
Intention of physicians to use SDM in practice	6	6	6	0.1
	Mean	Mean (SD)	Mean (SD)	*p*‐value
*How important do you find the attitudes below, and are these attitudes accomplished through SDM?*				
The patient is informed about important benefits and disadvantages of different treatment options	5.9	5.8 (0.9)	6.0 (1.0)	0.2
The background and relevant situation of the patient is discussed	5.7	5.5 (0.8)	5.8 (0.9)	0.1
The opinions and wishes of the patient are discussed during the treatment process	5.9	5.8 (0.7)	6.0 (0.8)	0.048
The decision for treatment is made together with the patient	5.9	5.9 (0.7)	5.9 (1.1)	NS
The patient is satisfied with the care process	5.9	5.8 (0.9)	5.9 (0.9)	NS
The patient is involved in the treatment process	5.8	5.7 (0.7)	6.0 (0.8)	0.1
The treatment chosen is appropriate for the specific patient	5.8	5.8 (0.8)	5.8 (1.2)	NS
**Total score for attitude**	**5.9**	**5.7 (0.6)**	**5.9 (0.7)**	**0.055**
*Subjective norm; how important are the opinions of these persons or social groups, and do they advise you to use SDM?*				
Colleagues	5.2	5.2 (1.0)	5.2 (1.1)	NS
Local residency training programme director	5.3	5.5 (1.0)	5.0 (1.5)	0.1
Insurers	2.6	2.3 (1.0)	3.0 (1.3)	0.002
Patients	5.0	4.9 (0.9)	5.0 (1.1)	NS
Health policy makers (e.g., national orthopaedic society, ministry of health)	3.9	3.7 (1.3)	4.1 (1.4)	0.08
**Total score for subjective norm**	**4.6**	**4.5 (0.7)**	**4.7 (0.7)**	**NS**
*Perceived behavioural control*				
I am convinced that I can share decision‐making in the clinic	5.9	5.8 (1.0)	6.0 (1.0)	NS
I have control over the level of SDM that is accomplished in the clinic	5.7	5.5 (1.0)	5.8 (1.2)	0.02
I can perform SDM without extending the duration of the consultation Time constraints are an important issue in SDM	3.6	3.3 (1.1)	3.9 (1.5)	0.036
Knowledge about SDM is important in order to perform SDM My knowledge about SDM is sufficient	5.4	5.2 (1.0)	5.5 (1.0)	0.027
Communication skills are important for SDM My communication skills required for SDM are sufficient	6.1	5.9 (0.7)	6.3 (0.7)	0.006
The patient is motivated to participate in SDM Patient motivation is important for SDM	5.1	5.0 (1.0)	5.1 (1.0)	NS
In general, the patient's knowledge, intelligence and understanding needed for SDM are sufficient Patient's knowledge, intelligence and understanding are important for SDM	3.7	3.7 (0.9)	4.2 (1.0)	0.046
**Total for behavioural control**	**5.3**	**5.2 (0.6)**	**5.5 (0.7)**	**0.021**

NS: not significant; SD: standard deviation; SDM: shared decision‐making; TPB: theory of planned behaviour

Of the three items of the TPB, attitude showed the highest scores, with a total score of 5.9. Although residents had a lower mean total score, there were no significant differences between the scores of residents and orthopaedic surgeons.

For subjective norm, the total score was 4.6. This was the lowest score of the three subdimensions. Of the factors of subjective norm, physicians viewed the opinion of the local residency training programme director as the most important influence. Low scores were given for the influence of health policy‐makers and insurers. Compared with residents, orthopaedic surgeons reported as being significantly more influenced by insurers in their SDM behaviour.

For perceived behavioural control, the total mean score was 5.3. Residents and orthopaedic surgeons gave high scores for the physicians' knowledge about SDM and for communication skills needed for SDM, and low scores for perceived control over time and for patients’ knowledge, intelligence and understanding. Residents scored significantly lower than orthopaedic surgeons on perceived behavioural control. The items on this dimension that received lower scores from residents were the level of control over SDM, time constraints, communication skills important for SDM, and patient knowledge, intelligence and understanding.

Furthermore, we saw that physicians older than 35 years had a higher total score on attitude (*p* = 0.036). No significant differences were seen between the scores of male and female physicians. In addition, the type of hospital (academic or non‐academic) was not associated with different scores for the items of the TPB.

### 3.2 Bivariate and multivariate analyses

In the bivariate analyses, attitude, subjective norm and perceived behavioural control were correlated with intention to engage in SDM (see Table 2). Of the three determinants, the one most strongly associated with intention to engage in SDM was perceived behavioural control. The determinant that was least associated with intention to engage in SDM was subjective norm.

**Table 2:** Bivariate analysis: Association with intention for SDM behaviour

Spearman's rho	Correlation	*p*‐Value
Attitude	0.45	<0.001
Subjective norm	0.28	0.001
Perceived behavioural control	0.53	<0.001

The variables that satisfied the criteria for entry into the multivariate analyses were higher attitude, higher subjective norm, higher perceived behavioural control and orthopaedic surgeon (professional role). Entry of these variables resulted in a model that explained 27% of the variance in the intention scores (R^2^ = 0.27, *p* < 0.001) (Table 3).

**Table 3:** Regression coefficients and 95% confidence intervals (CIs) of the intention for SDM behaviour

	**b (95%CI)**	**Standard error**	***p*‐value**
Coefficients			
Attitude	0.233 (−0.068, 0.534)	0.152	0.129
Subjective norm	−0.002 (−0.276, 0.271)	0.138	0.986
Perceived behavioural control	0.604 (0.291, 0,917)	0.158	<0.001
Professional role	0.081 (−0.228, 0.390)	0.156	0.607

SDM: shared decision‐making

## 4 DISCUSSION

The aim of the present study was to gain insight into the intention of orthopaedic residents and their supervisors to engage in SDM in the care of patients with hip and knee osteoarthritis. It showed that orthopaedic surgeons and residents generally express positive attitudes toward SDM in the care of hip and knee osteoarthritis patients. Lower scores were seen for perceived behavioural control and subjective norm. As expected, according to the TPB, the intention to engage in SDM was determined by attitude, subjective norm and perceived behavioural control. Intention to engage in SDM was most strongly associated with perceived behavioural control. In the resulting model, 27% of the variance in the scores on the intention to engage in SDM was explained by higher attitude, higher subjective norm, higher perceived behavioural control and the professional role “orthopaedic surgeon”.

Residents felt significantly less in control over factors influencing their SDM behaviour. Although, for all physicians, the mean scores for the physician's knowledge and skills relevant for SDM were high, residents were less confident that they possessed the communication skills needed to perform SDM, and they rated their knowledge about SDM lower than orthopaedic surgeons. This is a relevant finding as patient communication, and even SDM, have increasingly been implemented in medical education programmes in recent years. It is therefore to be questioned whether these pregraduation programmes have the desired effect. Additionally, the clinical experience of orthopaedic surgeons might be important in the control that these physicians experience regarding this behaviour.

External factors outside of the physician's perceived control contributed to the low score in perceived behavioural control. This resonates with other research findings, in which physicians experienced many barriers owing to external factors when implementing SDM (Gravel et al., 2006; Hofstede et al., 2013). In a review study by Gravel et al. (2006), the most important obstacles to implementing SDM mentioned by physicians were time constraints, characteristics of the patient and clinical context.

One of the external factors pointed out by physicians was that patients had limited abilities to participate in the decision‐making process. This is in line with a study by van der Horst and colleagues (2011). In this study, residents were more negative about the ability of patients to participate in decision‐making than their teachers. This perspective could partly be explained by physicians' (mis)interpretation of the concept of SDM. In SDM, the patient does not need to have medical expertise but needs to give information about his or her background, situation and preferences relevant for the medical decision. This in itself may be a challenge for some patients, but with coaching from the physician, most patients are keen to do this (Coulter & Collins, 2011). Even when patients have low health literacy, the level of SDM can be improved successfully by SDM interventions (Muscat et al., 2017).

Another important perceived obstacle is the extra time needed for SDM. In 2014, a review study investigated the effects of interventions to improve the adoption of SDM by healthcare professionals, and reported no difference in the duration of consultation after implementation of these interventions (Légaré et al., 2014), although it should be noted that most of the reviewed studies had no effect on the level of SDM. A Cochrane review on implementing decision aids reported a median increase in the duration of consultations of 2.6 min (Stacey et al., 2017). Little is known about the, possibly positive, effects of SDM on the total duration of healthcare provision—for instance, on the number of follow‐up visits to the outpatient clinic.

In our study, we found that physicians had high levels of attitude and competencies for SDM. By contrast, previous research on actual SDM behaviour in orthopaedics showed that there was much room for improvement (Frymoyer & Frymoyer, 2002; Woltz, Krijnen, Meylaerts, Pieterse, & Schipper, 2017). The difference between the physicians' positive scores on SDM in our study and the actual (relatively low) levels of SDM in the orthopaedic clinic could possibly indicate that physicians overestimate their SDM competencies, and that they may be unconsciously incompetent in this behaviour. This reasoning is supported by a review by Pollard, Bansback, & Bryan (2015), who described five studies that compared self‐reported attitudes about SDM with actual SDM behaviour. In most of these studies, the actual decision‐making behaviour appeared to be rather paternalistic, although most physicians had positive attitudes toward SDM (Pollard et al., 2015).

As mentioned, our study explained 27% of the variation in the intention to engage in SDM. This means that most of the variation in intention is explained by factors other than those included in our study. The level of variation in intention that was explained by the determinants of the TPB was in line with that found in other research. In the review by Thompson‐Leduc et al. (2015) on studies about SDM behaviour explained by the TPB, the predictability of the variance of intention varied, with R^2^ values ranging from 15% to 88%. Other mentioned factors explaining intention for SDM behaviour are self‐efficacy (Foy et al., 2007; Ten Wolde, Dijkstra, Empelen, Knuistingh Neven, & Zitman, 2008) and moral and professional norms (Daneault, Beaudry, & Godin, 2004; Godin et al., 2008; Sassen, Kok, & Vanhees, 2011; Thompson‐Leduc et al., 2015). In our study, the strongest predictor for SDM intention was perceived behavioural control, a finding which is not in line with the review by Thomas Leduc et al. (2015).

As the present study relied on self‐reported scores, it was susceptible to cognitive bias and socially desirable answers (de las Cuevas et al., 2012; Pollard et al., 2015). When attitude has been measured in interviews or focus groups, more salient beliefs and attitudes on SDM have been reported (Hajizadeh, Uhler, & Pérez Figueroa, 2015; Hofstede et al., 2013), and attitude scores have been found to be lower than in the present study.

We used closed‐ended items to measure the complicated construct of SDM based on the TPB. Our questionnaire was designed with the help of the manual developed by Francis and colleagues in 2004 (Francis et al., 2004). According to this manual, closed‐ended items are constructed by first executing a qualitative study which elicits commonly held beliefs about intention, attitude, subjective norm and perceived behavioural control. For SDM, extensive research is already available. Therefore, we did not execute this step, and based our items of the TBP on the current literature.

In our study, we had a response rate of 34%, which was comparable to that of other survey studies using the email approach (Yun & Trumbo, 2006). We approached the whole population of interest by email. For this, we used the email database of the Dutch Orthopaedic Society, which might not have been completely up to date. Selection bias might have occurred, as physicians with a positive attitude toward SDM might have been more inclined to participate in our survey.

A ceiling effect was seen in the questionnaire, with high median and mean scores for intention, attitude and perceived behavioural control.

The study indicated that the intention to perform SDM is high. As intention is correlated to actual behaviour (Ajzen, 1991; Daneault et al., 2004; Thompson‐Leduc et al., 2015), this finding provides indirect information about the clinical behaviour of physicians in caring for patients with hip and knee osteoarthritis. In our study, intention to engage in SDM was predicted by the three dimensions of the TPB, with perceived behavioural control having the strongest influence. Our findings imply that a shift towards positive attitudes about SDM has taken place in orthopaedic physicians.

However, these physicians, and particularly the residents, experience barriers to, and difficulties in, the implementation of SDM. The differences in perceived behavioural control between orthopaedic surgeons and residents underline the importance of incorporating SDM into the curriculum of medical students and postgraduates. Students and residents should be taught what SDM entails, and its impact. Furthermore, they should be aware of the various possibilities for implementing SDM efficiently, to overcome the perceived barriers. Information about current predictors of SDM behaviour among physicians working in the care of patients with hip and knee osteoarthritis is valuable and necessary for developing programmes that aim to improve SDM behaviour, as explained by the TPB (Conner, 2010).

## CONFLICTS OF INTEREST

The authors declare no conflicts of interest.

## Supporting information

Data S1. The questionnaire items of attitude, subjective norm and perceived behavioural control.
