# Supplementary material for: Experienced barriers in shared decision‐making behaviour of orthopaedic surgery residents compared with orthopaedic surgeons
Source: Musculoskeletal Care. 2019 Feb 27;17(2):198–205. doi: 10.1002/msc.1390 (PMC6850155; doi:10.1002/msc.1390)
Supplement: Supplementary file 1 — Data S1. The questionnaire items of attitude, subjective norm and perceived behavioural control. [file MSC-17-198-s001.docx]

**Supplement:**

| ATTITUDE | |
| --- | --- |
|  | - How important do you find that the patient is informed about important benefits and disadvantages of different treatment options - Implementing SDM entails that the patient will be informed about important benefits and disadvantages of different treatment options |
|  | - How important do you find that the background and relevant situation of the patient are discussed - Implementing SDM entails that the background and relevant situation of the patient will be discussed |
|  | - How important do you find that the opinion and wishes of the patient are discussed during the treatment process - Implementing SDM entails that the opinion and wishes of the patient will be discussed during the treatment process |
|  | - How important do you find that the patient is satisfied - Implementing SDM implies that the patient will be satisfied |
|  | - How important do you find that the patient is involved in the treatment process - Implementing SDM entails that the patient will be involved in the treatment process |
|  | - How important do you find that the treatment is suited for the specific patient - By implementing SDM the treatment will be tailored to the specific patient |
| SUBJECTIVE NORM | |
|  | - How important do you find the opinion of colleagues - Do colleagues advise you to use SDM |
|  | - How important do you find the opinion of the local residency training program director - Does the local residency training program director advise you to use SDM |
|  | - How important do you find the opinion of Insurers - Do insurers advise you to use SDM |
|  | - How important do you find the opinion of patients - Do patients advise you to use SDM |
|  | - How important do you find the opinion of health policy makers (e.g. national orthopaedic society, ministry of health) - Do health policy makers (e.g. national orthopaedic society, ministry of health) advise you to use SDM |
| PERCEIVED BEHAVIOUR CONTROL | |
|  | - I am convinced that I can apply SDM in decision making in the orthopaedic clinic |
|  | - I have control about the level of SDM that is accomplished in the clinic |
|  | - I can apply SDM without the consult taking longer - Time constraints are an important issue in SDM |
|  | - Knowledge about SDM is important in order to apply SDM - I have enough knowledge about SDM |
|  | - Communication skills are important for SDM - I have the communication skills required for SDM |
|  | - The patient is motivated to participate in SDM - Patient motivation is important for SDM |
|  | - In general the patient has enough knowledge, intelligence and understanding needed for SDM - Patient knowledge, intelligence and understanding is important for SDM |
